# To Conform or Not to Conform: Spontaneous Conformity Diminishes the Sensitivity to Monetary Outcomes

**DOI:** 10.1371/journal.pone.0064530

**Published:** 2013-05-17

**Authors:** Rongjun Yu, Sai Sun

**Affiliations:** School of Psychology and Center for Studies of Psychological Application, South China Normal University, Guangzhou, China; Cinvestav-Merida, Mexico

## Abstract

When people have different opinions in a group, they often adjust their own attitudes and behaviors to match the group opinion, known as social conformity. The affiliation account of normative conformity states that people conform to norms in order to ‘fit in’, whereas the accuracy account of informative conformity posits that the motive to learn from others produces herding. Here, we test another possibility that following the crowd reduces the experienced negative emotion when the group decision turns out to be a bad one. Using event related potential (ERP) combined with a novel group gambling task, we found that participants were more likely to choose the option that was predominately chosen by other players in previous trials, although there was little explicit normative pressure at the decision stage and group choices were not informative. When individuals' choices were different from others, the feedback related negativity (FRN), an ERP component sensitive to losses and errors, was enhanced, suggesting that being independent is aversive. At the outcome stage, the losses minus wins FRN effect was significantly reduced following conformity choices than following independent choices. Analyses of the P300 revealed similar patterns both in the response and outcome period. Our study suggests that social conformity serves as an emotional buffer that protects individuals from experiencing strong negative emotion when the outcomes are bad.

## Introduction

Humans are highly susceptible to social influence. When an individual's judgment conflicts with a group, the individual often conforms his judgment to that of the group [1]. This ubiquitous phenomenon that individuals change their behaviors and attitudes to match the majority's behavior is known as social conformity [2], [3]. Depending on individual's intrinsic motives behind their behavior, there are two main types of conformity. If people rely on others to determine what is correct to do in uncertain contexts, it is referred as informational conformity. In other situations, if people adjust their behaviors in order to ‘fit in’ with the majority, this underlying form of social influence is called normative conformity [3], [4] Informational conformity is concerned with accuracy and the search for information about reality, whereas normative conformity is concerned with social interaction [4]. In normative conformity, individuals may not change their own opinions but simply change their behavior under social pressure [5].

Several accounts of conformity have been proposed with respect to different types of conformities [6]. The accuracy account of informative conformity posits that individuals often refer to social information to gain an accurate understanding of reality and effectively respond to social situations, especially during times of uncertainty [5]. Previous memory research showed that individuals may conform to information supplied by a group of confederates when reconstructing their memories for stimuli [7]. Investigators, for example, demonstrated that when faced with unfamiliar songs, a common strategy to find the best is to choose the most downloaded one [8]. However, informative conformity is not necessarily effective since people can conform to poor targets and what the majority believe is not always correct. The affiliation theory of normative conformity proposes that individuals are often engaged in more conscious and deliberate attempts to gain the social approval of others, building rewarding relationships with them in the process. Individuals are frequently rewarded for behaving in accordance with opinions of the majority [3]. Using a mental rotation task, Berns et al. studied conforming behavior in face of wrong information and found that mismatches or conflicts between one's own preference and the others' motivate him to switch individual choice towards the consensus [1]. Previous research also demonstrated that disagreement between the subject and the confederates induced stronger conflicts monitoring [9], but decreased activity in reward regions such as the nucleus accumbens [10]. The above research provided neural support for the normative conformity, which suggested that affiliation with others is rewarding and people tend to follow others for the diminished conflicts or social deviance.

Furthermore, the anterior cingulate cortex (ACC) receives projections from the midbrain dopaminergic regions and has been proposed to play an important role in reward processing, error detection and conflict monitoring. Recent Event-related potential (ERP) studies have identified two components, the feedback related negativity (FRN) and the P300. FRN is suggested to be generated at the anterior cingulate cortex by source localization analysis and preforms a role in encoding reward prediction error, conflict detection and error monitoring [11]. The FRN is a negative deflection at fronto-central recording sites and peaks between 250–300 ms post onset of outcome feedback [12]. A stronger negativity in amplitude predicts a stronger FRN effect. Previous studies revealed that the FRN is more negative to monetary losses compared with monetary gains, and is more negative for ‘worse than expected’ prediction error than prediction congruence [12]–[16]. The deviation from the initial prediction is termed as prediction error or expectation violation [17]. Additionally, a recent research demonstrated that feedbacks indicating deviance from the group norm elicited a feedback-related negativity, a brainwave signal known to be elicited by objective performance errors and losses. The results imply that the brain treats deviance from social norms as an error [18]. Moreover, another reward related ERP component named the P300 peaks around 300–600 ms after stimulus presentation and has the most positive deflection at posterior electrode locations. The P300 effect is stronger when the waveform is more positive. It is suggested that the P300 is sensitive to a later, top-down controlled process of outcome evaluation, where factors related to the allocation of attentional resources come to play. Those factors include reward valence, reward magnitude, and magnitude expectancy [19].

Given the evidence that the FRN encodes both prediction error signal and attitude conflicts, and previous study also demonstrated that individuals treat deviance from social norms as prediction errors or cognitive conflicts, we predict that the FRN can encode both informative and normative conformity signals and the FRN amplitude would be modulated by the degree of conformity. However, in more primitive times, we conformed to others not just for information or social approval, but for emotional comfort. Berns's study also suggested that independence was associated with increased amygdala and caudate activity, which provided the first biological evidence for the involvement of perceptual and emotional processes during social conformity [1]. Additionally, another study by Berns et al. demonstrated that the tendency to change one's evaluation of a song was positively correlated with activation in the anterior insula and anterior cingulate [20], two regions that are frequently associated with physiological arousal and negative affective states [21].

Taking the above studies together, we predict that going against the group was more unpleasant or conflicting whereas going with the group was more rewarding or acceptable. People tend to conform to others not just for reference to others' information or social approval of others, but for pursuit of positive emotion when affiliated with others or aversion of negative emotion when conflicted with others. Thus, humans have evolved to value conformity (i.e., by insula, amygdale or cingulate such neural mechanisms) which may function as buffers against physical and emotional pains. However, there is no existing theory illustrating this hypothesis. Additionally, previous studies mainly focused on normative conformity in which individuals have to choose whether to follow the crowd or not in the presence of normative pressure and few studies have investigated spontaneous conformity. Here, we designed a group gambling task where the participants, together with another two confederates, were required to choose either the left or right field and received win or loss feedback regarding each person's outcome. When participants' choices turned out to be different from others' decisions, we referred this condition as independent condition; when the two confederates chose different fields, we termed this condition as baseline; if participants' choice was the same as the other two confederates' choices, the situation is termed as conformity condition. Two critical manipulations were introduced in our experiment. First, participants were required to make decisions before seeing others' choices, which minimized the normative pressure. It was still possible that participants can learn the dominant preference based on observations of previous trials, and this learned ‘social norms’ can put pressure on participants' current choice. However, such pressure was much less than the pressure people normally face when choosing after explicitly knowing others' choices. Second, the confederates chose one field (dominate field) more frequently than the other while the probability of winning a reward in any field was at a 50% chance level. We can't preclude that the participants still show informational conformity from probabilistic nature of the confederates' choices. They can subjectively learned the informative rules from perceived choices of others, even if these choices provided no informational or accurate reference theoretically. Additionally, they may also refer to others though there was no any monetary incentive to follow others' choices.

Our aim was to test the mentioned new hypothesis of social conformity, and further analyze whether social conformity modulates the neurophysiological representation of the experienced monetary outcomes using event-related potentials (ERP) techniques. We hypothesized that, at the behavioral level, if participants conformed to others, they chose the predominant field more frequently and experienced more pleasure. At the neural level, during the response period, if participants' choices were different from others, enhanced FRN and diminished P300 would be observed since being independent was associated with more emotional conflicts; during the outcome period, when individuals conformed to others, their sensitivity to monetary rewards (e.g. the actual gain or loss) would be reduced at the outcome stage, which can be reflected in the reduced loss-win FRN difference waveform and P300 difference waveform.

## Materials and Methods

### Participants

Among the twenty-four electroencephalo-graph (EEG) participants, three participants stated that they completely disbelieved the experimental manipulation in the interview after the EEG test. These participants were excluded from data analysis, leaving twenty-one participants (10 male, mean age ± SD, 20.23±1.27years) for the following analysis. All participants were right-handed, and had normal or corrected-to-normal vision, and were screened for neurological or psychiatric disorders. The study was approved by the Academic Committee of the School of Psychology at South China Normal University. All participants gave written, informed consent and were informed of their right to discontinue participation at any time. All participants were paid a uniform amount (¥40, about 7 US dollar) for their participation.

### Experimental paradigm

Before the EEG experiment, participants were told that they would attend a group gambling task with the other two partners on the internet. The purpose of this task was to study how people make decisions in a group. Unbeknown to participants, the two partners were confederates. Then participants were asked to stand against the wall and a picture of him/her was taken using a digital camera. Similarly, two pictures of the confederates (matched on gender) were taken in the presence of the participant respectively. The purpose of using participants' and two confederates' photos was to make the experimental setup more realistic which induced the participants to believe they were playing with two real persons. After that, both the participant and the confederates were given a comprehensive description of the tasks they would perform and the real participant was directed into the electroencephalo-graph room while the two confederates were directed into other two different experiment rooms. The same confederates were used for all participants in this study (two males for the male participants and two females for the female participants).

**Figure 1:**
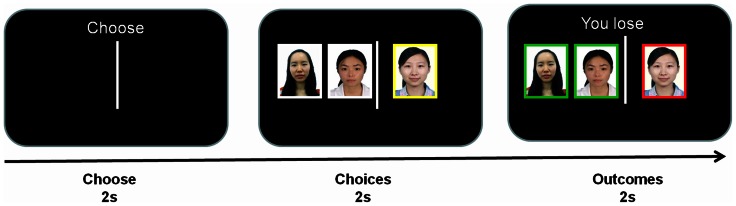
Experimental procedure. At the beginning of each trial, a vertical line separating the screen into the left and the right, was presented along with a text ‘Choose’. Participants were required to choose either the left or the right field by pressing the corresponding key within 2 seconds. They were told that the other two participants were also making decisions at the same time. Then, the choice of the participant and the choices of the two confederates in separate rooms were revealed on the screen for 2 seconds. Each individual's choice was indicated by the location of his/her photo and the participant's choice and two confederates' choices were highlighted in different colors. Then they received a “You win” or “You lose” feedback for 2000 ms. The colors (green or red) associated with positive/negative feedback were counterbalanced. The given trial represents an independent-loss condition in which the subject has chosen the right field and loses whereas the two confederates have chosen the left field and won. The subjects of the photographs have given written informed consent, as outlined in the PLOS consent form, to publication of their photographs. The images used in the figure are not the original images used in the study, but similar images used for illustrative purposes only.

At the beginning of each trial, a vertical line separating the screen into the left and right two parts, was presented along with a text ‘Choose’ (see Fig.1). Participants were required to make an alternative choice (indicating a monetary reward) from either the left or the right field by pressing the corresponding key within 2 seconds. They were told that the other two participants were also making decisions in the same task. Then, the choice of the participant in the EEG room and the choices of the two confederates in separate rooms were revealed on the screen for 2 seconds. Each individual's choice was indicated by the location of his/her photo (3.5°high, 5.5°wide in visual angle, white against a black background) and the participant's choice and two confederates' choices were highlighted in different colors respectively, which makes it easier to distinguish their own choices from the other two confederates'. For example, if the participant chose left, his/her photo would be presented in the left field, highlighted in yellow; if one confederate chose right, his/her photo would be shown in the right field, highlighted in white. Unbeknown to our participants, the confederates' choices had been arranged ahead of the experiment using the following criteria: in 70% trials, the two confederates chose the left field; in 15% trials, they chose the right field; in the remaining 15%, one confederate chose the left field and the other chose the right one. The probability of choosing left/right field was counterbalanced between participants. Thus, overall, the two confederates chose on field much more frequently than the other field and participants can learn this ‘social norm’ during the experiment. Then participants received a “You win” in green or “You lose” in red feedback for 2000 ms. The outcomes for the two confederates were also revealed and highlighted in the corresponding colors. They were told that they got an opportunity to choose one trial from the whole trials and their extra reward was based on the outcome of the selected one. If they received a “You win” feedback, they would be rewarded another ¥20 after finishing the task, otherwise, they would get ¥0. Unbeknown to participants, the win/loss outcomes were predetermined according to a pre-specified sequence, with the chance of winning in either the left or the right field was always 50%. The order of experimental trials was pseudorandom that with the constraint of no more than 3 consecutive trials with the same type of experimental condition. The experiment consisted of two blocks of 120 trials each.

At the end of the experiment, participants were debriefed and required to indicate how satisfied they felt for each type of feedback using a 11-point analogue Likert scale (0 =  not at all, 10 =  very intensely). There were six experimental conditions at the outcome stage, i.e. independent loss (IL): chose to be different from others and actually lost; independent win (IW): chose to be different from others and actually won; baseline loss (BL): chose to conform to one of the confederates and actually lost; baseline win (BW): chose to conform to one of the confederates and actually won; conformity loss (CL): chose to conform to others and actually lost; conformity win (CW): chose to conform to others and actually won). They were also asked to indicate how surprised they felt when receiving these outcomes.

### ERP Recording and Analysis

The participant was seated comfortably about 1.5 m in front of a computer screen in a dimly lit and electromagnetically shielded room. The experiment was administered on a Lenovo computer in CRT display, with 1024*768 resolutions, using E-prime software to control the presentation and timing of stimuli. The EEG was recorded from 64 scalp sites using tin electrodes mounted in an elastic cap (NeuroScan4.5) according to the International 10–20 system. The vertical-oculogram (VEOG) was recorded from left supra-orbital and infra-orbital electrodes. The horizontal electro-oculogram (HEOG) was recorded from electrodes placed 1.5 cm lateral to the left and right external mastoid. All electrode recordings were referenced to an electrode placed on the left mastoid, and the impedance was maintained below 5ΚΩ. The EEG and electro-oculogram (EOG) were amplified using a 0.05–70 Hz band-pass and were continuously sampled at 500 Hz/channel for off-line analysis.

The EEG data were re-referenced off-line to linked mastoid electrodes by subtracting from each sample of data recorded at each channel one-half the activity recorded at the right mastoid. Ocular artifacts were corrected with an eye-movement correction algorithm [22]. Epochs of 800 ms (with 200 ms pre-stimulus baseline) EEG for each electrode were time-locked to the onset of choice and feedback stimuli and were sorted by experimental conditions. The FRN and P300 were filtered using a 20 Hz low-pass. After that they were baseline corrected by subtracting from each sample the average activity of that channel during the baseline period. All trials in which EEG voltages exceeded a threshold of ±70 µν during the recording epoch were excluded from analysis. All trials in which EEG voltages exceeded a threshold of ±70 µν during the recording epoch were excluded from analysis.

The grand average FRN amplitudes were measured in a window of 200 to 400 ms after the onset of choice and outcome respectively. The peak value of the P300 was detected as the most positive value in the 300–500 ms time window. We focused on the FRN responses at the anterior frontal midline electrodes (Fz) and the P300 responses at the posterior midline electrode (Pz), since the FRN and P300 effects were the largest at these electrodes, respectively. The FRN data were entered into one way ANOVA with three different conditions (independent, baseline vs. conformity) for the response period, and repeated-measures ANOVAs with conditions (independent, baseline vs. conformity), feedback (win vs. loss) as two within subject factors for the outcome period. (The six conditions were as follows. IL: chose to be different from others and actually lost; IW: chose to be different from others and actually won; BL: chose to conform to one of the confederates and actually lost; BW: chose to conform to one of the confederates and actually won; CL: chose to conform to others and actually lost; CW: chose to conform to others and actually won). The Greenhouse–Geisser correction for repeated measures was applied where appropriate.

## Results

To determine whether participants showed a general conformity effect, we first analyzed the percentage of choosing the left dominant field. Compared with the 50% probability of gaining a reward randomly, independent-sample t-test results showed that participants were more likely to choose the field dominantly chosen by the two confederates (mean ± SD, 52.27%±4.37%), t (20)  = 54.53, p<0.001, suggesting that participants tended to unconsciously follow others' choice, though the chance of winning a reward in that field was not informative.

### Response locked FRN

**Figure 2:**
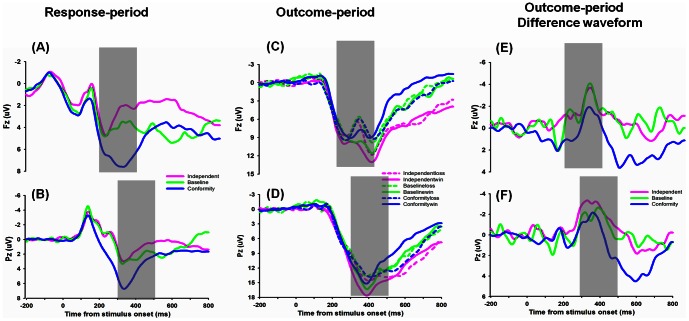
The ERP grand-average waveforms. Grand-average waveforms at channel Fz & Pz for experimental conditions (independent, baseline vs. conformity) in the response period (A&B) and six experimental conditions (IL: choose to be different from others and actually loss; IW: choose to be different from others and actually win; BL: choose to conform to one of the confederate and actually loss; BW: choose to conform to one of the confederate and actually win; CL: choose to conform to others and actually loss; CW: choose to conform to others and actually win) in the outcome period (C&D). During the outcome period, we also found a significant linear interaction effect, reflecting that the sensitivity to monetary reward was modulated by conformity degree. So we further drawn the difference waveforms for three conditions (independent loss-win, baseline loss-win, and conformity loss-win) (E&F). The shaded 200–400 ms time window was for the average calculation of the FRN effects. The P300 was measured as the most positive value in the 300–500 ms time window.

**Figure 3:**
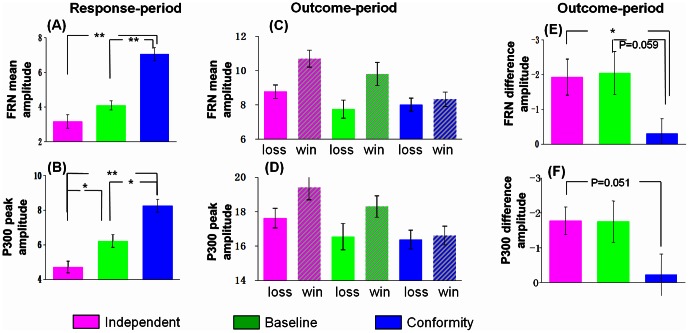
The amplitudes of the FRN and P300. The FRN and P300 amplitudes (mean, SE) for three experimental conditions (Independent, Baseline vs. Conformity) (A&B) during response period and six experimental conditions (IL: choose to be different from others and actually loss; IW: choose to be different from others and actually win; BL: choose to conform to one of the confederate and actually loss; BW: choose to conform to one of the confederate and actually win; CL: choose to conform to others and actually loss; CW: choose to conform to others and actually win) (C&D) during outcome period, and the difference waveform for three conditions (independent loss-win, baseline loss-win, conformity loss-win) were shown (E&F). ^**^p<0.01, ^*^p<0.05.

**Figure 4:**
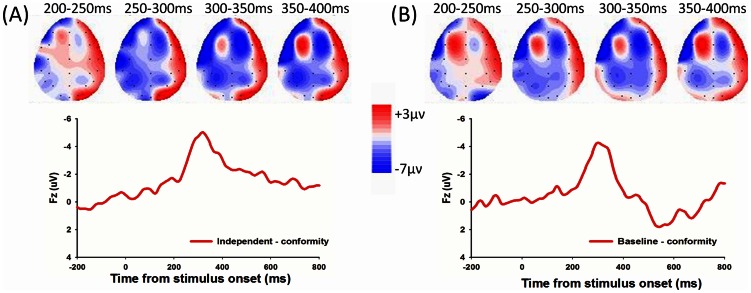
The FRN effect in the response period and topographical maps. (A)The independent minus conformity difference waveform and corresponding topographical maps (200–400 ms, 50 ms interval) and (B) the baseline minus conformity difference waveform and corresponding topographical maps (200–400 ms, 50 ms interval) were shown.

The ERP grand-average waveforms at channel Fz for 3 conditions (independent, baseline vs. conformity) during the response period were shown in Fig.2A. We found a main effect of choice type (independent, baseline, and conformity), F (2, 20)  = 24.011, p<0.001, and a significant linear main effect, F (2, 20)  = 30.887, P<0.001. Post-hoc analysis revealed a significant difference between independent and conformity choices, with enhanced FRN amplitude for independent (mean ± SE, 2.97 µν ±0.90) than conformity (mean ± SE, 7.03 µν ±0.84), t (20)  = −5.558, P<0.001(see Table 1). We also found a significant difference between baseline and conformity choice, with more negative FRN amplitude for baseline (mean ± SE, 3.87 µν ±0.83) than conformity, t (20)  = −6.020, P<0.001. There was no significant difference between independent and baseline, t (20)  = −1.598, P = 0.126(see Fig.3A). These results suggest that the FRN encodes different choice types, with most positive FRN amplitudes when conforming to others. Additionally, the FRN effect of independent minus conformity and corresponding topographical maps were shown in Fig.4A, and the FRN effect of baseline minus conformity and corresponding topographical maps were shown in Fig.4B.

**Table 1:** The subjective ratings, FRN and P300 amplitude during the response period.

	Independent	Baseline	Conformity
FRN amplitude (µν)	2.97±0.90	3.87±0.83	7.03±0.84
P300 amplitude (µν)	4.82±0.91	6.10±0.95	8.22±1.02

### Feedback locked FRN

The ERP grand-average waveforms at channel Fz for 6 experimental outcomes during the outcome period were shown in Fig.2C. A repeated-measures ANOVAs on the average FRN amplitudes, with choice types (independent, baseline vs. conformity) and the feedback valence (win vs. loss) as within factors, revealed a significant main effect of choice types, F (2, 20)  = 3.336, p = 0.049, a significant main effect of valence, F (2, 20)  = 8.251, p = 0.009, and a marginally significant interaction effect, F (2, 20)  = 2.831, p = 0.084(see Fig.3B). The FRN amplitudes were shown in Table 2. Since the degree of conformity changes linearly from independent, baseline to conformity condition, we also examined the linear interaction effect of FRN and found an obvious linear interaction effect, F (2, 20)  = 7.889, p = 0.011, suggesting that the FRN was modulated by the degree of conformity.

**Table 2:** The subjective ratings, FRN and P300 amplitude during the outcome period.

	Independent-loss	Independent-win	Baseline-loss	Baseline-win	Conformity-loss	Conformity-win
Satisfaction rating	3.14±0.63	8.95±0.33	4.14±0.47	7.57±0.46	3.38±0.51	9.05±0.37
Surprise rating	6.00±0.56	5.86±0.66	5.43±0.34	3.81±0.56	7.33±0.40	3.86±0.66
FRN amplitude (µν)	8.72±1.18	10.82±1.19	7.78±1.38	9.86±1.37	8.10±1.19	8.28±1.05
P300 amplitude (µν)	17.62±1.37	19.35±1.28	16.20±1.22	17.99±1.05	16.18±1.00	16.16±0.96

**Figure 5:**
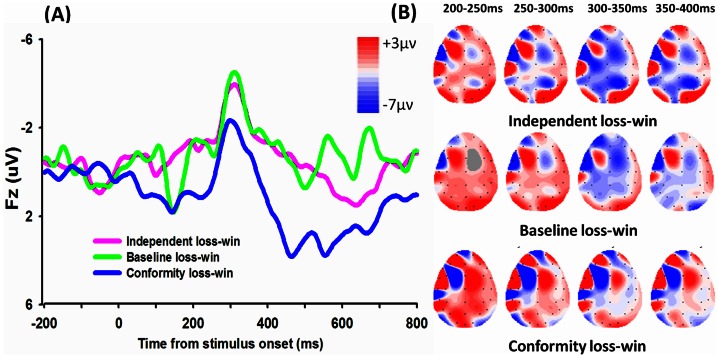
The difference waveform in three conditions for outcome period and topographical maps. (A)The difference waveforms (loss-win) in three conditions (independent, baseline and conformity) and (B) the corresponding topographical maps (200–400 ms, 50 ms interval) were shown.

The significant linear interaction effects suggested that the sensitivity to monetary outcomes (loss-win FRN) were modulated by the degree of conformity, which was from independent, baseline to conformity(see Fig.2E). Paired-sample t test revealed a significant main effect on the loss-win FRN difference waveform between independent and conformity choices, with a more negative FRN amplitude for independent (mean ± SE, −2.10 µν ±0.62) than conformity (mean ± SE, −0.18 µν ±0.50), t (20)  = −2.809, P = 0.011, a marginally significant difference between baseline (mean ± SD, −2.08 µν ±0.99) and conformity condition, t (20)  = −2.002, P = 0.059, and no significant difference between baseline and conformity condition, t (20)<1 (see Fig.3E).The FRN difference waveform (loss minus win) in three conditions (independent, baseline vs. conformity) (see Fig.5A) and the corresponding topographical maps (see Fig.5B) were shown.

### Response locked P300

The ERP grand-average waveforms at channel Pz for three conditions (independent, baseline vs. conformity) during the response period were shown in Fig.2B. One-way ANOVA on the peak amplitudes of the P300, with conditions (independent, baseline vs. conformity) as independent factors, revealed a main effect of conditions, F(2, 20) = 14.915, p<0.001, a significant linear main effect, F(2, 20) = 29.589, P<0.001, suggesting that the P300 was sensitive to different social choices. For the three different conditions, Post-hoc analysis revealed a significant difference between independent and conformity choice, t (20)  = −5.440, p<0.001, with a more negative FRN amplitude for independent (mean ± SE, 4.82 µν ±0.91) than conformity (mean ± SE, 8.22 µν ±1.02). The P300 amplitude was shown in Table 1. We also found a significant difference between baseline (mean ± SE, 6.10 µν ±0.95) and conformity choice, t (20)  = −3.13, p = 0.005, and a significant difference between independent and baseline condition, t (20)  = −2.209, p = 0.039 (see Fig.3B).

### Feedback locked P300

The ERP grand-average for six experimental outcomes and difference waveforms (loss minus win) at channel Pz during the response period were shown in Fig.2D&F. A repeated-measures ANOVAs on the peak amplitude of the P300, with conditions (independent, baseline vs. conformity) and the actual outcomes (win vs. loss) as independent factors, revealed a main effect of conditions, F (2, 20)  = 4.586, p = 0.017, suggesting that the P300 was modulated by choice types. No significant main effect of outcome valence was found, F (2, 20)  = 2.371, p = 0.139, reflecting that the P300 may not be sensitive to the actual monetary losses. No significant interaction effect was found between conditions and valence, F (2, 20)  = 2.316, p = 0.122(see Fig.3D). The specific P300 amplitude was shown in Table 2.

### Correlation analysis

**Figure 6:**
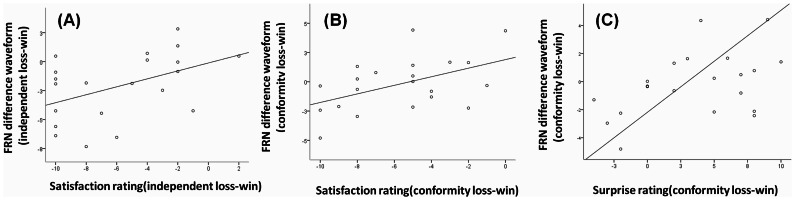
The correlation between subjective satisfaction/surprise rating and the FRN difference waveform. For the outcome period, the correlations between subjective satisfaction rating and FRN difference waveform (independent loss-win, conformity loss-win) were shown separately (A&B). The correlation between subjective surprise rating and FRN difference waveform (conformity loss-win) was also shown (C).^**^p<0.01, ^*^p<0.05.

The scatter plots for correlation between subjective satisfaction/surprise ratings and the FRN difference waveforms were shown in Fig.6A–C. The post-experiment subjective rating questionnaires were applied for scaling the participant's feelings towards win/loss outcomes following independent, baseline and conformity choice. We also showed the specific ratings in Table 2 during outcome period. We found that the FRN effect (loss minus win) was significant correlated with the self-reported satisfaction difference (loss minus win) in independent condition, r = 0.458, p = 0.037(see Fig.6A) and in conformity condition, r = 0.493, p = 0.023(see Fig.6B); but not in the baseline condition, r = 0.205, p = 0.372, suggesting that the FRN was stronger linked with subjective evaluation of outcomes after social salient choices (independent or conformity). Additionally, in conformity condition, we also found a significant correlation between the FRN effect (loss minus win) and self-reported surprise difference (loss minus win), r = 0.447, p = 0.042(see Fig.6C). No other significant correlation involving the FRN was found, p>0.05.

## Discussion

In the present study, we demonstrated that social conformity occurs even when little normative pressure and informative instructions were present. Using a group gambling task combined with ERP techniques, we found an enhanced FRN and a diminished P300 when individual's choices were different from others, suggesting that being independent is stressful and aversive. During the outcome stage, the loss minus win FRN effect was significantly reduced in conformity condition than in independent or baseline condition, suggesting that following the crowd reduces the emotional impact of negative outcomes.

In our study, one critical manipulation is that participants made their own choices before knowing others' choices. Unlike previous studies in which participants had to make choices in the presence of group opinions (i.e. normative pressure), our manipulation reduced individuals' responsibility of being different from others. Moreover, the group choices were probabilistic, further reducing the social pressure to conform. Since the group opinions were ambiguous and unbeknown before individuals' decisions, there was little incentive to gain social approval or avoid social conflicts. Additionally, in the present study, the probability of gaining a reward was at a 50% chance level, the dominated field choice was no better than alternative choice and provided no useful information to individuals. Thus, our behavioral conformity results are difficult to be explained neither by the affiliation account of normative conformity nor the accuracy account of informative conformity. It is possible that social conformity may be implicitly generated by normative pressure (normative conformity) and information about perceived accuracy of certain choices (informational conformity). In our study, the purpose was not to exclude the influence of normative pressure, but to minimize the explicit normative pressure, since participants were required to make choices spontaneously before seeing the choices of others. Additionally, we can't ensure that the participant doesn't subjectively learn the rules from perceived choices of others, even though this perceived choices provided no informational or accurate reference theoretically. However, we still found the conformity effect at the behavioral level; at the neural level, the sensitivity to monetary outcomes was modulated by the degree of conformity. Thus, this social influence on outcome processing or evaluation leads to other theoretical interpretations about the FRN.

The mainstream theory of the FRN holds that it reflects the activity of reinforcement learning system which continually evaluates ongoing events against the expected outcomes, and encodes prediction errors that guide our decision making by signalling the need for adjustment of behaviour in the future [23]. During the response period, we found the most positive FRN when conforming to others, since participants may regard their difference from others as a prediction error. However, the FRN can also be evoked by negative emotions due to the experienced conflicts or errors when made predictions. During the outcome period, the FRN can be elicited by prediction error or monetary losses since the participants may obtain negative feedbacks conflicted with their initial predictions. Nevertheless, this FRN effect can also be evoked by negative emotions involved with expectancy violation or negative outcomes. Also, source localization analysis demonstrated that the FRN, which is likely to be generated in the ACC, plays a crucial role in conflicts detection, performance monitoring and behavioral adjustment [9]. In our study, participants were required to make spontaneous response, and the explicit normative conflicts were minimized. Thus, this FRN effect was elicited not only by normative conflicts but also by other alternative components, e.g. emotion, motivation. Additionally, the FRN is not limited to error processing and general performance-monitoring: it also acts as part of affective-motivational system [24]. During the response period, we further tested the correlation between FRN waveform when making independent choices and conformity degree, revealing a non-significant effect, r = 0.312,p = 0.168. Though this effect was not obvious and cannot demonstrate that the FRN discouraged individuals to make independent choices adequately, we attributed this non-significance to the non-sufficient number of samples. During the outcome stage, we investigated the difference waveform (loss-win) in different conditions. Results showed that the loss minus win FRN effect was significantly reduced in conformity condition than in independent or baseline condition, suggesting that following the crowd reduces the sensitivity to monetary outcomes. Also, this reduced FRN difference waveform when conforming to others had a positive correlation with changes in participants' subjective ratings of satisfaction (conformity loss-win), and the enhanced FRN difference waveform during independent condition also positively correlated with changes in participants' subjective ratings of satisfaction (independent loss-win), indicating that the emotional evaluation indexed by the actual outcomes direct the motivational reactions to ongoing events [25]–[27]. We can presume that individuals conform to others not only for the accuracy of information and social affiliation with others, but also for risk aversion or negative emotion avoidance.

We propose that the reduced emotional sensitivity to negative outcomes following conformity may explain the herding behavior in our experimental context. On the one hand, we argue that affiliation with others reduced individual's sensitivity to monetary outcomes and enhanced their subjective satisfaction ratings. Thus, conformity can be considered as a positive reinforcement and protects us from experiencing strong negative emotion when the outcomes are bad. On the other hand, this reduced sensitivity to negative outcomes drives people to make more conformity behaviors because individuals have a tendency to avoid negative emotions or seek positive outcomes. Additionally, we also found a significant correlation between surprise rating during independent condition (independent loss-win) and FRN difference waveform (independent loss-win), informing that both individual's satisfaction and surprise levels are correlated with FRN changes and direct the motivational choices. We argue that individual also has a tendency to seek novel stimuli besides positive ones. Berns's study has provided the first biological evidence for the involvement of perceptual and emotional processes during social conformity [1]. Additionally, a recent fMRI study similar to social conformity suggests that individuals are more willing to choose defaults (similar to unconscious conformity) to avoid the enhanced negative emotion associated with choosing non-defaults (similar to non-conformity) [21]. Taken together, we can postulate that emotion or motivation may play an important role in human's herding behaviors as well. Conformity is a decision with low risk and protects individuals from experiencing strong negative emotion when the outcomes are bad.

Here, we propose an emotion buffer hypothesis, which argues that the FRN does not reflect the cognitive processes of evaluating performance or detecting prediction errors, but rather, reflects the processes of assessing the motivational/affective impact of social reactions (like conforming to others) or outcome events (like losing money) [12], [24], [28]. In our study, we confirmed that following the crowd (even individuals were not aware of that strategy) can reduce the experienced negative emotion even when the group decisions turn out to be a bad one. Individuals learn this social behavior by assessing or anticipating the reduced negative emotional impact after herding, and conformity serves as an emotional buffer that protects them from experiencing strong negative emotions when the outcomes are unsatisfactory. Thus, it is possible that social conformity is not only driven by normative pressure or accurate information, but also by anticipating the reduced emotional impact after herding according to our emotion buffer hypothesis.

For the P300, the results also revealed a main effect of choice types with the most negative amplitude during independent condition but the most positive amplitude when following the crowd both in the response period and outcome period. However, we found no significant effect of valence on the P300, which is consistent with Yeung's and Sato's findings that the P300 is sensitive to reward magnitude but insensitive to reward valence [29], [30]. It is generally believed that P300 is implicated in a large number of cognitive and affective processes and is traditionally associated with allocation of mental resources [31]. Researcher also found that the modulated P300 amplitudes evoked during decision and outcome evaluation tasks probably reflect the evaluation of the functional significance of the feedback stimuli [15]. Moreover, early studies reported that the P300 is related to multiple evaluative processes and the larger P300 amplitude is usually elicited by stimuli with high emotional value, informative feedback stimuli, and target stimuli [32], [33]. In our study, individuals need more mental resources to evaluate outcomes and make decisions from the possible rules, and work with more satisfactory ratings when conforming to others. Thus, conformity can be more informative or positive, which can also be reflected on the P300.

To sum up, we demonstrate a behavioral conformity effect when little explicit normative pressure is presented and little informative group opinions are available. Conformity choices activate diminished FRN and enhanced P300, compared with independent choices, suggesting that conformity itself is rewarding or positive. During the outcome stage, the loss minus win FRN effect is significantly reduced in conformity condition than in independent or baseline condition and this reduction has significant correlation with the subjective ratings. These results suggest that social conformity servers as an emotional buffer that protects individuals from experiencing strong negative emotion when the outcomes are bad.
